# Tamm Plasmon Resonance as Optical Fingerprint of Silver/Bacteria Interaction

**DOI:** 10.1021/acsami.3c05473

**Published:** 2023-06-01

**Authors:** Simone Normani, Pietro Bertolotti, Francesco Bisio, Michele Magnozzi, Francesco Federico Carboni, Samuele Filattiera, Sara Perotto, Fabio Marangi, Guglielmo Lanzani, Francesco Scotognella, Giuseppe Maria Paternò

**Affiliations:** † Center for Nano Science and Technology@PoliMi, Istituto Italiano di Tecnologia, Via Raffaele Rubattino, 81, 20134 Milano, Italy; ‡ Biomedical Engineering Department, Politecnico di Milano, Piazza Leonardo Da Vinci, 32, 20133 Milano, Italy; § SuPerconducting and Other INnovative Materials and Devices Institute (SPIN), Consiglio Nazionale delle Ricerche (CNR), Corso F.M. Perrone 24, 16152 Genova, Italy; ∥ Dipartimento di Fisica, Università di Genova, via Dodecaneso 33, 16146 Genova, Italy; ⊥ Physics Department, Politecnico di Milano, Piazza Leonardo Da Vinci, 32, 20133 Milano, Italy

**Keywords:** photonic crystals, Tamm plasmon, silver nanostructures, bacterial responsivity, bacterial detection

## Abstract

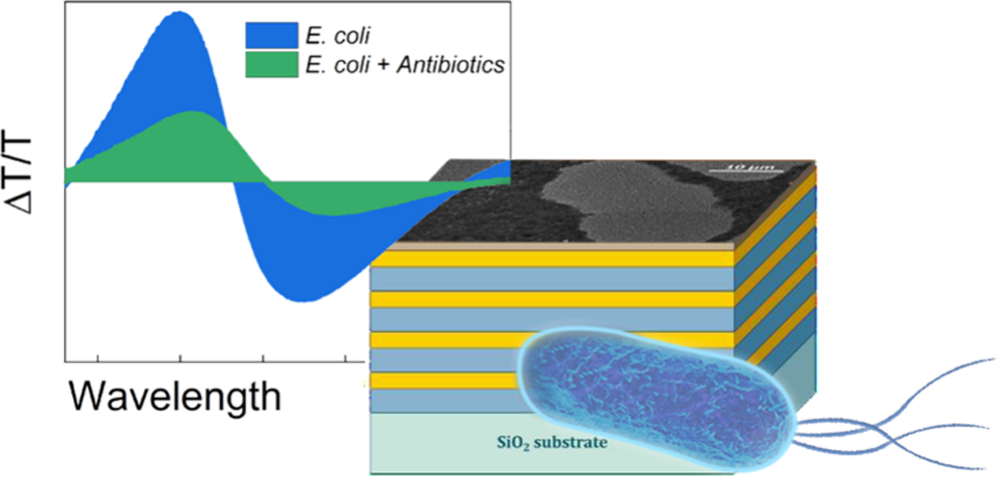

The incorporation of responsive elements into photonic crystals is an effective strategy for fabricating active optical components to be used as sensors, actuators, and modulators. In particular, the combination of simple multilayered dielectric mirrors with optically responsive plasmonic materials has proven to be successful. Recently, Tamm plasmon (TP) modes have emerged as powerful tools for these purposes. These modes arise at the interface between a distributed Bragg reflector (DBR) and a plasmonic layer and can be excited at a normal incidence angle. Although the TP field is located usually at the DBR/metal interface, recent studies have demonstrated that nanoscale corrugation of the metal layer permits access to the TP mode from outside, thus opening exciting perspectives for many real-life applications. In this study, we show that the TP resonance obtained by capping a DBR with a nanostructured layer of silver is responsive to *Escherichia coli*. Our data indicate that the modification of the TP mode originates from the well-known capability of silver to interact with bacteria, within a process in which the release of Ag^+^ ions leaves an excess of negative charge in the metal lattice. Finally, we exploited this effect to devise a case study in which we optically differentiated between the presence of proliferative and nonproliferative bacteria using the TP resonance as a read-out. These findings make these devices promising all-optical probes for bacterial metabolic activity, including their response to external stressors.

## 1 Introduction

Distributed Bragg reflectors (DBRs) are simple multilayered systems in which a photonic band gap (PBG) arises from the periodic arrangement of materials with different refractive indices, in analogy with the well-known phenomenon of reflection of X-rays by crystals. Although DBRs have been mostly employed for fundamental studies and/or for building up passive optical components, in the last couple of decades, the introduction of selected responsive elements has paved the way to the effective utilization of DBRs as simple yet effective tools for the active manipulation of light and for sensing purposes.^1−5^

Plasmonic materials are indeed well-established responsive elements, as their optical and electronic properties depend on the environment and on the application of external stimuli.^6^ For instance, the integration of dynamically tunable plasmonic materials in photonic crystals has proven beneficial for color display technology: here, the photonic component ensures high optical purity and stability even in miniaturized structures, while the plasmonic counterpart allows for high switching speed.^7,8^ Focusing on DBRs, such a plasmonic/photonic coupling can occur via the incorporation of plasmonic nanostructures as an integral part of the multilayer. In this way, the modification of the plasmon frequency and the complex dielectric function that is achieved via electro/photodoping permits an active manipulation of the PBG and thus of the optical read-out.^3,4,9−11^

Recently, we have introduced plasmonic materials as a capping layer in DBRs, with the aim to exploit hybrid plasmonic/photonic optical modes at their interface and with the goal of demonstrating their responsiveness to external stimuli such as bacteria. Photonic-based devices may offer a low-cost and portable alternative to traditional methods for microbial detection, which is of urgent need due to the emergence of antibiotic resistance and food spoilage caused by bacterial contaminants.^12^ In this context, photonic-based devices can be in principle portable and easy-to-read alternatives to traditional state-of-the-art methods.^12^ In our first attempt, we incorporated a thin layer of silver (8 nm) on top of DBRs, relying on the well-known capability of silver to interact with bacteria, an effect that in turn induces marked changes in the structural, morphological, and optical properties of the metal.^13,14^ Although we could observe a clear PBG blue shift upon exposure to bacteria in our initial studies (∼10 nm, accompanied by a strong broadening),^15,16^ we realized that this originated simply from a change in the refractive index conditions experienced by the DBR, without the excitation/involvement of any well-defined plasmonic resonance. Thus, our sensing information was essentially photonic and encoded entirely on the broad PBG feature (full width half-maximum, FWHM, exceeding largely 100 nm), rendering the read-out difficult to be interpreted.

In any case, these results prompted us to the investigation of Tamm plasmons (TPs) which are electromagnetic modes confined between a DBR and a noble metal layer (i.e., gold or silver).^17−19^ The TP mode can be experimentally observed as a narrow resonance peak (dip) in the transmission (reflection) spectrum of a sample at wavelengths within the band gap of 1DPC.^20^ In contrast to localized surface plasmons resonances, TPs are polarization independent and can be excited with high quality factor at normal incidence angle, without the need for phase-matching techniques like a grating or prism coupling.^20,21^ Beside this advantageous operative feature, the relative simplicity of the planar structure necessary to achieve the TP resonance lends itself to both facile fabrication (i.e., spin casting or sol–gel deposition^22,23^) and large-scale fabrication procedures. So far, TPs have been utilized for many purposes, including lasing,^24^ modification of light-matter interaction,^24−27^ radiative coolers,^28^ thermal emitters,^29^ and optical sensors,^22,30−33^ among others. However, this comes with a disadvantage, as the electric field distribution of the TP mode is located predominantly at the DBR/metal interface, thus being almost inaccessible to external stimuli. This would limit greatly the operational possibilities of such devices, i.e., for sensing applications and for electrochromic devices. Interestingly, very recently it has been demonstrated that patterning or corrugation of the metal film at the micro/nanometer level enables both reduction of metal losses and exposition of the TP field to external stimuli, such as changes of the refractive index.^34^ Taken together, these are crucial advantages for the development of responsive systems and sensors, as such a resonance mode can be as narrow and, possibly, stimuli-responsive as Fabry–Perot cavity modes^18^ while ensuring direct access to external probes.

Here, we exploit such an effect for the purpose of bacterial detection and for the monitoring of their proliferative status. In particular, we observe that the TP mode originating at the interface between a nanostructured film of silver and a DBR is highly responsive to exposure to bacteria, as highlighted by a blue shift and a clear damping of the TP resonance as compared with the control measurements. Our data indicate that such a modification of the TP mode can be ascribed to the silver–bacteria interaction, which likely causes a change of Ag charge carrier density owing to the silver ions uptake by bacteria, as we have demonstrated in a recent study.^13^ Electrodoping experiments confirm that corrugation at the nanoscale plays a significant role in the detection mechanism, as, in this case, the TP field enhancement is exposed to the air interface. To take advantage of these features, we devised a proof-of-concept experiment, in which we tested the capability of such TP devices to discriminate between proliferative and nonproliferative bacteria. Intriguingly, we cannot observe significant bacterial-induced changes on the TP resonance when we rendered bacteria nonproliferative via kanamycin or chloramphenicol administration. This can hold promise for the application of TP devices as simple optical drug screening platforms.

## 2 Materials and Methods

### 2.1 Fabrication of TP Devices

Silica (SiO_2_) and titania (TiO_2_) were the materials chosen for the fabrication of the colloidal photonic crystals^35^ due to their high transparency in the visible range, their relatively high refractive index mismatch, and their availability. To center the PBG around 650–700 nm, keeping into account an expected porosity of the layers around 20–30%, the thickness of the dielectric layers should be about 110–130 nm for SiO_2_ and 70–80 nm for TiO_2_. These values were estimated using a DBR simulation program based on the transfer matrix formalism and using the Maxwell Garnett model for glass layer porosity.^36^ DBRs were then fabricated by spin casting deposition. We selected this method because of its simplicity and availability of the instrumentation. Note that although in colloidal photonic crystals, interlayer and intralayer roughness is relatively high, this can be taken as an advantage for various applications that require infiltration of selected analytes and probes.^2,37−44^ In our case, we reckon that roughness can be beneficial for depositing a corrugated silver layer, which eventually allows access to the TP mode from the outside. However, such a method comes with a disadvantage, as it relies on the manual sequential deposition of the silica/titania layers from the colloidal dispersions, implying that this technique leads inherently to a large batch-to-batch variability in terms of spectral response (see for instance Figure 5a,c). The fabrication starts with the preparation of the colloidal dispersions: a 30 wt% aqueous solution of SiO_2_ nanoparticles (*Ludox*, average diameter = 40 nm) was diluted with distilled water to yield a concentration of 6 wt %, and a TiO_2_ nanoparticle powder (*GetNanoMaterials*, average diameter = 5 nm) was dissolved in distilled water to prepare a 10 wt % aqueous solution. These concentrations were estimated to obtain the required thickness of SiO_2_ and TiO_2_. The two solutions were then processed via a tip sonicator (*Branson 450 Digital Sonifier*) to homogenize them and break apart particle clusters, and then filtered with 0.22 μm filters to remove the remaining clusters. The DBRs were then prepared by depositing four pairs of alternating SiO_2_ and TiO_2_ layers on 2 cm squares of microscope glass, dropping 150 μL of solution per layer, spinning the sample at 2000 rpm with a spin coater for 1 min, and annealing it on a hot plate at 350 °C for 20 min after every layer deposition. Finally, a metal layer was deposited on the top of the DBR by thermal evaporation (thickness 20 nm). For most of the experiments, we deposited a silver layer, whose thickness was optimized to improve TP resolution and achieve low FWHM. In additional experiments, such as electrodoping and control measurements with bacteria, we used gold as a metal layer. Electrodoping experiments were carried out following the same experimental procedure used in our previous work.^11^

### 2.2 Optical Spectroscopy and Scanning Electron Microscopy

The visible and near-infrared (Vis–NIR) transmission and reflection measurements were performed on the samples before and after exposure to the bacterial cultures, using a deuterium halogen lamp (*AvaLight-D(H)-S*) and a fiber-coupled spectrometer (*Avantes, AvaSpec-HS2048XL-EVO*), averaging over 30 measurements with an integration time of 2 ms. The measured data were normalized to the spectrum of a reference blank sample (silica glass slide for transmission, silver mirror for reflection) and a dark baseline. Reflection measurements in particular were averaged over 5 different spots across each sample.

Imaging of the sample surfaces was performed using scanning electron microscopy (SEM, *Tescan*) with various degrees of magnification, so as to assess the presence of *E. coli* and the state of the bacterial cells in contact with both plain glass and silver-coated surfaces.

### 2.3 *E. coli* Cultures and Sample Exposure

Microorganisms used for experiments are from the 25922 strain of *Escherichia coli* and 14028 strain of *Salmonella enterica* (provided by *ATCC*). Luria–Bertani (LB) broth and LB agar were used, respectively, for liquid culture and on-plate growth assays. The liquid bacterial cultures were grown overnight in LB medium in an incubator at a constant temperature of 37 °C, with a 200 rpm agitation rate. They were then diluted to OD_600_ 0.5 before moving them to LB agar plates. Each sensor was then exposed on an agar plate to a volume of 100 μL of liquid culture and left at 37 °C overnight. For sensor tests involving antibiotics, cultures were diluted to OD_600_ 0.5 and later exposed to kanamycin A (C_18_H_36_N_4_O_11_, 50 μg/mL in water) or chloramphenicol (C_11_H_12_N_2_O_5_Cl_2_, 170 μg/mL in ethanol) for 12 h. Note that we employed the terms “proliferative” and “proliferative state” throughout the text to refer to the active metabolic states of the cell population, while nonproliferative subsets are instances related to dead or inhibited cells after antibiotics or bacteriostatic treatment, respectively. In turn, active metabolic processes (including protein turnover, lipids synthesis, or, in general, metabolites exchange with the external environment) are directly involved in the interaction with the metal surface of the device.

### 2.4 Optical Simulations

The simulations were performed by means of the WVASE software (J.A. Woollam Inc.). The optical model consisted of a stack of dielectric layers, each characterized by its own thickness and complex dielectric function. Fresnel boundary conditions at the interface between the layers were assumed. For the simulation of the DBR backbone, the model (bottom to top) included (i) a silica substrate, (ii) 4 identical repetitions of a nanoporous silica layer, and a nanoporous titania layer. The nanoporous layers were modeled as Bruggeman effective media composed of a silica (titania) backbone and voids, as already proven appropriate for these systems.^45^ The dielectric functions of the silica and the titania backbone were taken from refs (46) and (47), respectively. During the fit, the dielectric functions of the backbones were kept constant, and only the thickness and the void fraction of the porous layers were left free to vary.

## 3 Results and Discussion

### 3.1 Device Morphology and Optical Properties

**Figure 1:**
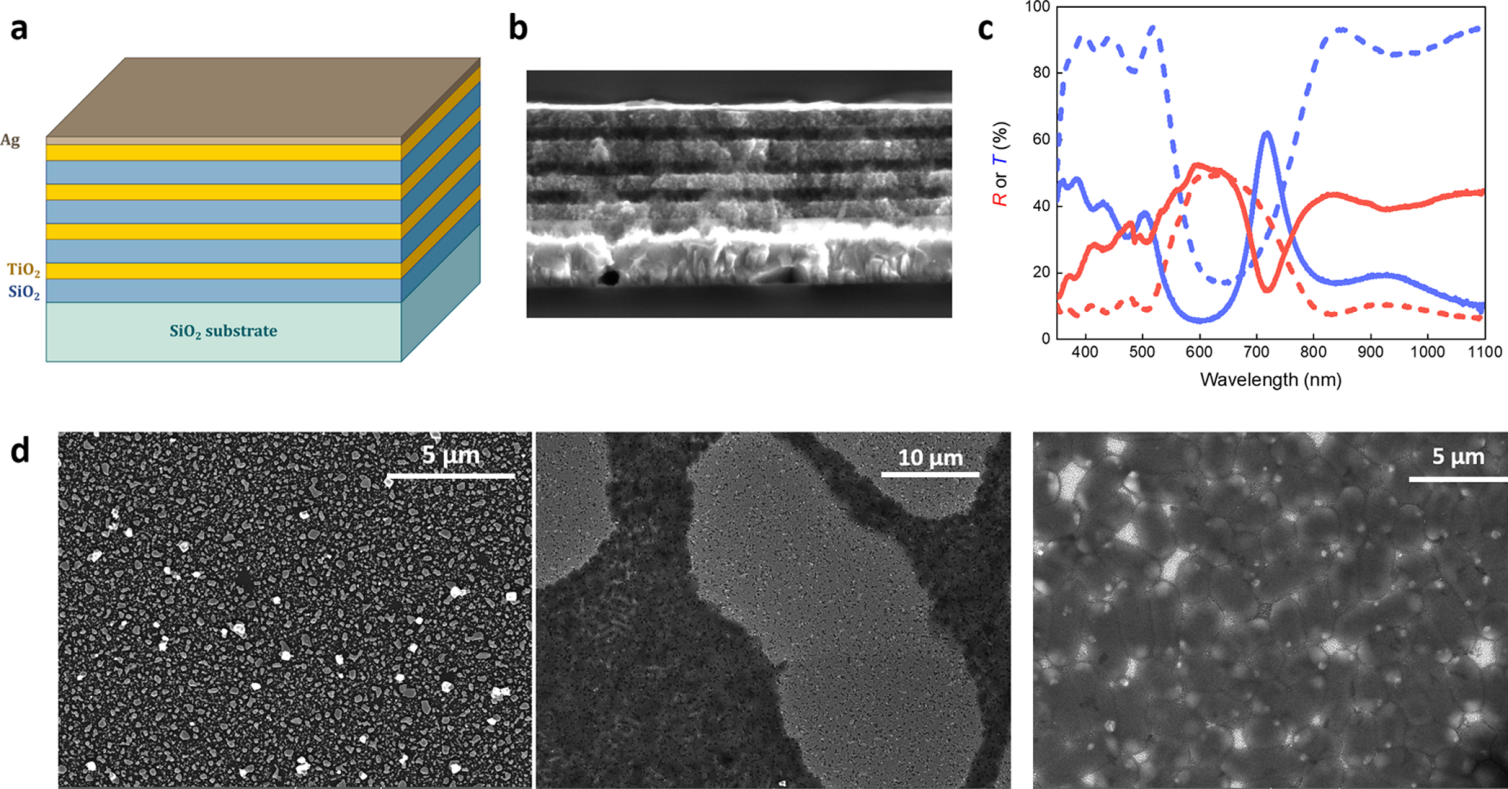
Structure, morphology, and optical read-out of the TP device. (a) Sketch of the DBR with the Ag capping layer. (b) SEM cross section of the multilayered system. We worked out a layer thickness of 130/80 nm for silica/titania and a capping layer of about 20 nm. Optical simulations reproduced with a good degree of approximation of the spectral feature of such devices, with a thickness of 110/70 nm and a capping layer of 30 nm (see Section 2 and the Supporting Information Section). (c) Reflection and transmission spectra of a typical sample, before (dashed lines) and after (solid lines) Ag deposition, showing the rise of the Tamm plasmon resonance around 725 nm. (d) SEM images of the Ag layer before (left panel), after *E. coli* exposure (center panel), and zoom on the bacterial colonies to highlight the formation of Ag cluster inside the cells.

A sketch of the DBR and its cross section are presented in Figure 1a,b, respectively. It consists of four bilayers of silica/titania deposited via spin coating from their colloidal nanoparticle suspensions, capped with a 20 nm layer of Ag deposited on the top of the DBR via thermal evaporation. Such a thickness ensures the emergence of the TP mode while permitting the characterization of the device also via transmission measurements (Figure S1). The transmission/reflection spectrum clearly reports a PBG located at 550–750 nm (Figure 1c). The addition of the Ag layer increases the overall reflectivity of the samples across the visible range and leads to the rise of a relatively narrow transmission peak (and a corresponding drop in reflection) positioned close to the PBG low-energy edge at around 725 nm. This peak can be ascribed to the emergence of the TP resonance.^22^

Scanning electron microscopy images on the capping Ag layer (Figure 1d) reveal a film that is composed of an assembly of nanoplates in close contact with each other exhibiting irregular shapes and sizes (average radius = 42 nm, average thickness = 20 nm, Figure S2). The nanostructured Ag film has an important role here due to three important reasons: (i) it leads to the appearance of the TP spectral feature. This is considerably narrower than the PBG (FWHM = 27 vs 138 nm), implying that its intensity and position is a relatively sensitive read-out upon exposure of analytes; (ii) the intrinsic and high bio-responsivity of nanosilver, which results in bacteria-driven modifications of silver plasmonic properties;^13,14^ and (iii) the corrugation at the nanoscale that does not hamper the emergence of the TP resonance while allowing direct access to its field, as demonstrated in a recent publication.^34^ Hence, our main idea is to exploit all these three advantages to build up an optical sensor capable of detecting the presence of bacteria, as well as being able to map out their metabolic activity with the broad view to develop drug-testing platforms.

Exposure of the device to *E. coli* colonies in an Agar plate leads to the formation of extended bacterial communities on the top of the surface (Figure 1d), with the bright spots located in close proximity to the bacterial membrane that can be linked to the uptake and incorporation of Ag^+^ ions clusters inside the cells.^48−50^ The brightness of these clusters can be due to an increase in secondary electrons related to the local membrane deformation.^49^ Such a mechanism is commonly recognized as the main driving force of nanosilver/bacteria cytotoxicity.^51^ Bacterial death can be evaluated preliminarily from cell morphology, which for healthy *E. coli* cells is usually rod-shaped rather than circular.^49^ Further, the presence of a well-defined zone of inhibition in the contaminated Agar medium can be linked to the eradication of bacteria, confirming the biocidal activity of the Ag layer (Figure S3).

### 3.2 Exposure to *E. coli* Modifies TP Resonance

**Figure 2:**
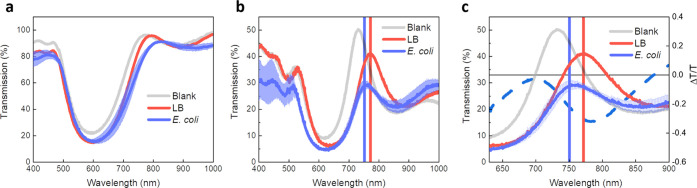
Effect of bacterial exposure on TP devices. (a) Transmission spectra of pristine silica/titania multilayers (blank) and exposed to LB and *E. coli* cells. We take both transmission and reflection measurements, while for simplicity sake, we show only transmission data in the main text. All the measurements involving bacterial cells were taken on biological triplicates. The curve represents the average over those measurements, and the shadow is the standard deviation. (b) Transmission spectra of TP devices exposed to LB and *E. coli*. (c) Zoom on the TP resonance spectral region, highlighting the 22 nm blue shift and decrease of transmission of the TP state upon contamination with bacteria. The dashed line represents the differential spectrum (Δ*T*/*T*) calculated as (*T*_E. coli_ – *T*_LB_)/*T*_LB_, which highlights the modification of the spectral response (both shifts and changes in transmission) of the TP resonance when exposed to bacteria.

We then proceed to the evaluation of the effects of bacterial exposure on the plasmonic/photonic spectral response. In general, our experimental routine consisted in a control measurement, in which the device was exposed only to the culture medium (Luria-Bertani broth), followed by actual exposure to the LB medium contaminated with *E. coli* cells. To confirm that the bio-responsive element of our system is indeed represented by the silver layer, we first carried out measurements on a bare DBR (Figure 2a). In this case, we observe a broadening of the PBG owing to the infiltration of the medium in the porous structure, without any clear difference between the samples exposed to LB and LB/bacteria. After having assessed that the bare DBRs cannot detect clearly the presence of bacteria, we then passed to the evaluation of TP devices. Here, we see strong modifications of the spectral features upon contamination with bacteria (Figure 2b) when compared to the LB-treated sample, namely, (i) a broadening of the PBG (FWHM = 160 vs 128 nm, for *E. coli* and LB, respectively) that we attribute to the inhomogeneous population of bacteria on the top of the silver layer, which increases the degree of optical disorder (refractive index) at the interface with air; and (ii) a clear blue shift (22 nm) of the TP and damping of its transmission (20%). To highlight these observations, we plotted the differential transmission spectra (Δ*T*/*T*) for LB and *E. coli*, which is essentially the difference between the spectrum of the perturbed sample (LB/bacteria) and the control sample (LB), normalized to the transmission of the control (dotted line in Figure 2c). Specifically, the negative signal centered at around 760 nm accounts for both Tamm mode blue-shift and decrease of transmission; thus, it can be taken as a diagnostic spectral parameter for identifying the presence of bacteria. Note that the blue shift and decreased transmission observed here have the same physical origin of the spectral changes observed recently in the plasmon resonance of nanostructured silver layers upon contamination with *E. coli*.^13^ By combining ultrafast optical spectroscopy, X-ray diffraction, and imaging, we assigned those changes to the silver oxidative dissolution in the presence of bacteria.^14^ In this work, we enhance the detection sensitivity by transducing the bacterial effect into a relatively narrow TP resonance. To be quantitative, the blue shift of the silver plasmon resonance upon bacterial exposure divided by its FWHM lies around 5%,^13^ whereas for our previous silver/DBR devices (without Tamm resonance), the PBG blue-shift/FWHM ratio amounts to 7%.^15^ On the other hand, interestingly, the blue shift/FWHM ratio for the Tamm resonance is more than one order of magnitude higher than in the abovementioned cases (80%), suggesting that this approach can afford a relatively high sensing capability.

We also assessed the capability of our hybrid device to monitor the presence of a different bacterium, namely, *S. enterica*. In analogy with *E. coli*, this is a Gram-negative bacterium of pharmacological and medical interest due to its pathogenicity. Again, we observe a ∼20 nm blue shift of the TP mode (Figure S4), suggesting that our observations are somehow general, at least for bacteria sharing the same cellular envelope.

### 3.3 Metal Corrugation Affects TP Response to Exogenous Stimuli

**Figure 3:**
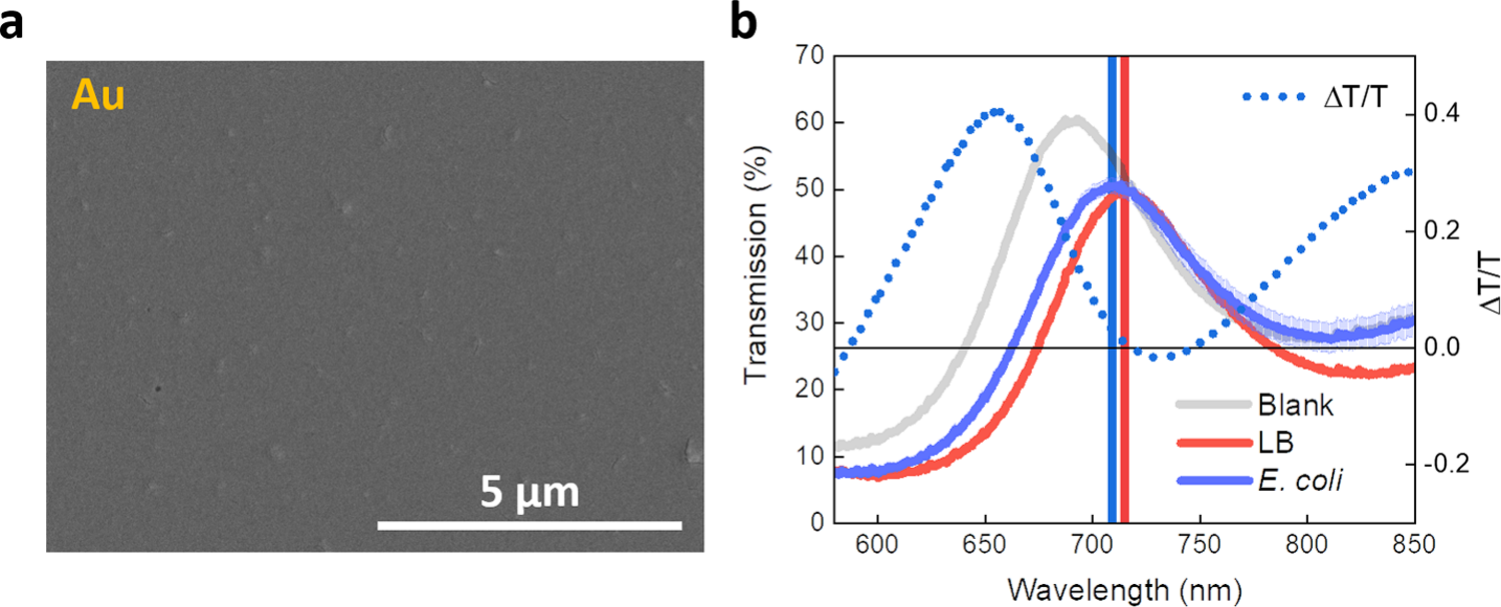
Effect of bacterial exposure on gold-based TP devices. (a) SEM image of the Au layer (thickness 20 nm, obtained via thermal evaporation). (b) TP resonance of the device exposed to *E. coli* cells. Also in this case, we report the differential transmission to highlight the difference between the perturbed sample and the control (dashed line).

To evaluate the role of the nanoscale corrugation of our films on the accessibility of the TP field, we fabricated and characterized TP devices with gold as a metallic plasmonic layer. We selected gold owing to its biocidal activity^52^ while ensuring a well-defined TP resonance.^22^ This metal layer (20 nm) appears as a compact film, contrary to what is observed in silver (Figure 3a). We thus proceeded to the evaluation of the bio-responsivity of such a TP device by exposing it to *E. coli*, following the same protocol adopted for silver-based systems. In this case, the blue shift was clearly less prominent than in the silver-based TP device (5 nm), while we could not note any substantial decrease of transmission. We reckon that this can be attributed to the following physical and chemical differences between silver and gold: (i) decreased bioactivity in gold due to the relatively small surface/volume ratio in smooth layers. Roughness is one of the most important features necessary for achieving effective biocidal activity, as the surface available for ionic release and bacterial uptake decrease considerably passing from nanostructured/rough to bulky/smooth materials.^53,54^ In addition, the smooth and compact gold layer would limit greatly the accessibility of the TP field for external probes and analytes;^34^ (ii) the lower reactivity of gold than that of silver, i.e., against oxidative dissolution. This implies that gold produces fewer ions than silver, which can be eventually uptaken by bacteria.^55^

**Figure 4:**
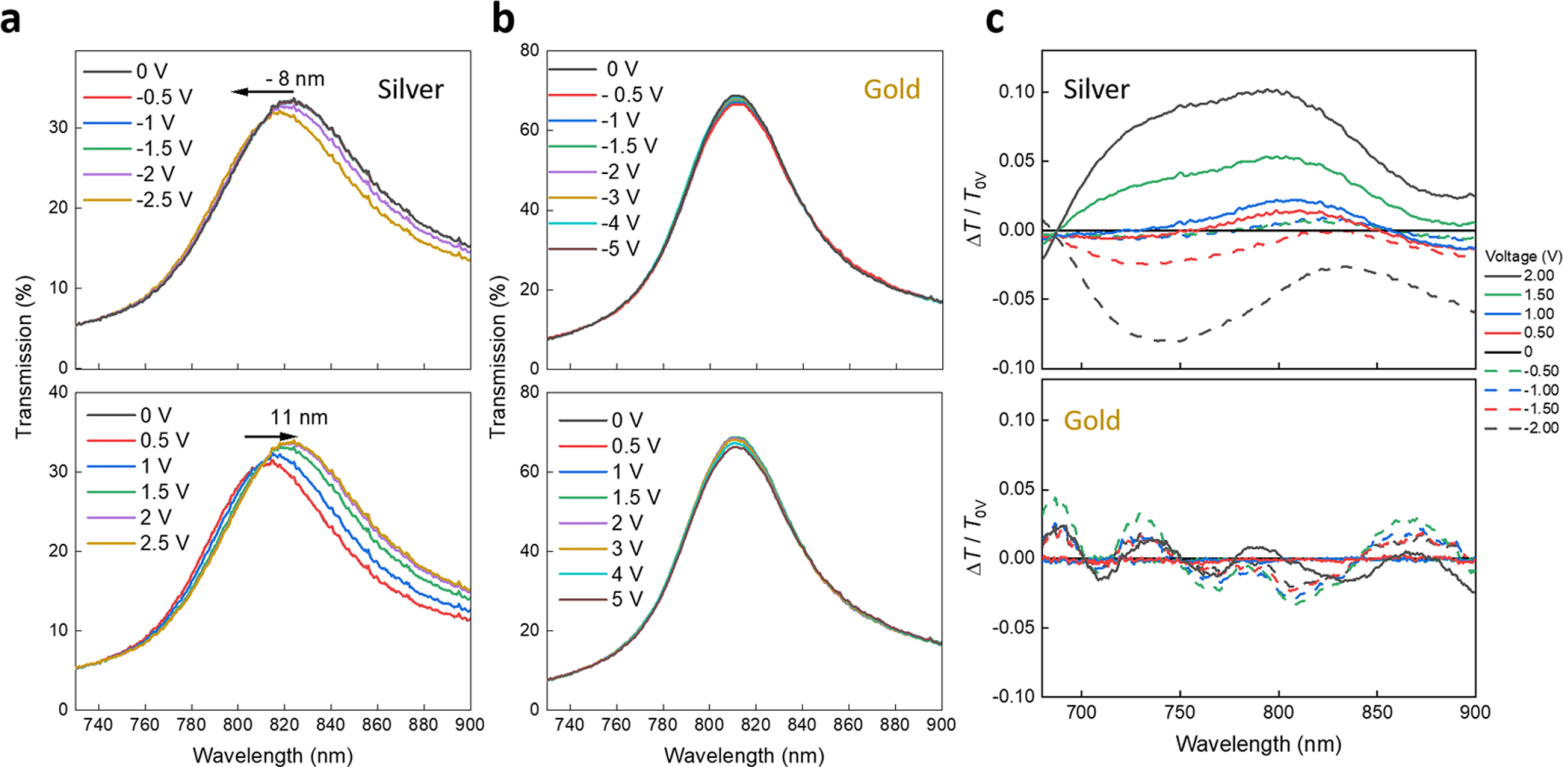
Electro-responsivity of Ag- and Au-based TP resonance. (a) Transmission of the TP resonance in the silver-based device upon application of negative (top) and positive (bottom) voltage. (b) Transmission of the TP resonance in the gold-based device upon application of negative (top) and positive (bottom) voltage. (c) Differential transmission spectra upon application of positive and negative bias for silver- (top) and gold-based TP devices.

We then proceed to an additional experiment, aiming at disentangling these two phenomena concurring to the less effectiveness of gold-based TP than silver. To this end, we set up an electrodoping experiment, by taking inspiration from our recent works on electro-responsivity of DBRs.^9,11,56^ Specifically, we fabricated our TP device on an indium tin oxide (ITO) electrode and sandwiched it with a second ITO layer on the top to provide an electrical connection (Figure S5). We then applied a negative or positive DC voltage to the composite structure and looked at the transmission spectrum in real time. The transmission spectra show a clear shift of the TP peak as a function of applied voltage: the peak undergoes a red shift (11 nm) and increase of transmission under positive bias, while we observe a blue shift (8 nm) and a decrease of transmission under negative bias (Figure 4a). As briefly mentioned above, we proposed that the bacterial-driven modification of the silver plasmon resides on the release of positive metal ions, within the scheme of silver oxidative dissolution.^13,14,51^ This can be essentially rationalized as a “biological doping”,^15^ as removal of Ag^+^ will leave behind an excess of negative charge. The results plotted in Figure 4 are thus fully consistent with this interpretation, for both phenomena, lead to an accumulation of negative charges in the plasmonic metal. In addition, these results confirm that the TP field can be indeed accessed from the outside, provided that metal is corrugate/structured.

We verified this by performing the same experiment on smooth gold-based devices (Figure 4b). Here, we could not observe any significant effect on TP peak position, while we noted a small decrease/increase of transmission (2%) upon application of a negative/positive bias. To highlight the higher magnitude of electric-responsivity of the silver TP than the gold-based resonance, we plotted the differential transmission of these two systems (Figure 4c). These results confirm that metal corrugation essentially dictates accessibility of the TP field from the outside, rendering this resonance highly appealing for building up sensors, actuators, and modulators. Interestingly, by means of optical modeling, we were able to reproduce both the occurrence of the TP mode and its blue shift upon the increase of electron density hence corroborating the experimental data. All of the details about modeling and simulated spectra can be found in the Supporting information section and in Figure S6.

### 3.4 TP Resonance Probes Proliferative Status of Bacteria

Since the uptake of Ag^+^ ions by bacteria is driven by their metabolic processes, for instance, those controlling expression of nucleophile-containing proteins (i.e., thiols and amino groups),^57,58^ one would expect a relationship between the modulation of TP resonance and their proliferative status. In general, the link between metabolic activity and antibiotic/drug bacterial uptake has been investigated deeply,^59^ while in our case, it would allow building up optical sensors for bacterial metabolic activity, including their response against external stressors (i.e., drugs and antibiotics), which rely on the easy-to-assess TP read-out. To verify such a hypothesis, we then devised a proof-of-concept experiment in which we exposed the devices to both proliferative and nonproliferative *E. coli* cells.

**Figure 5:**
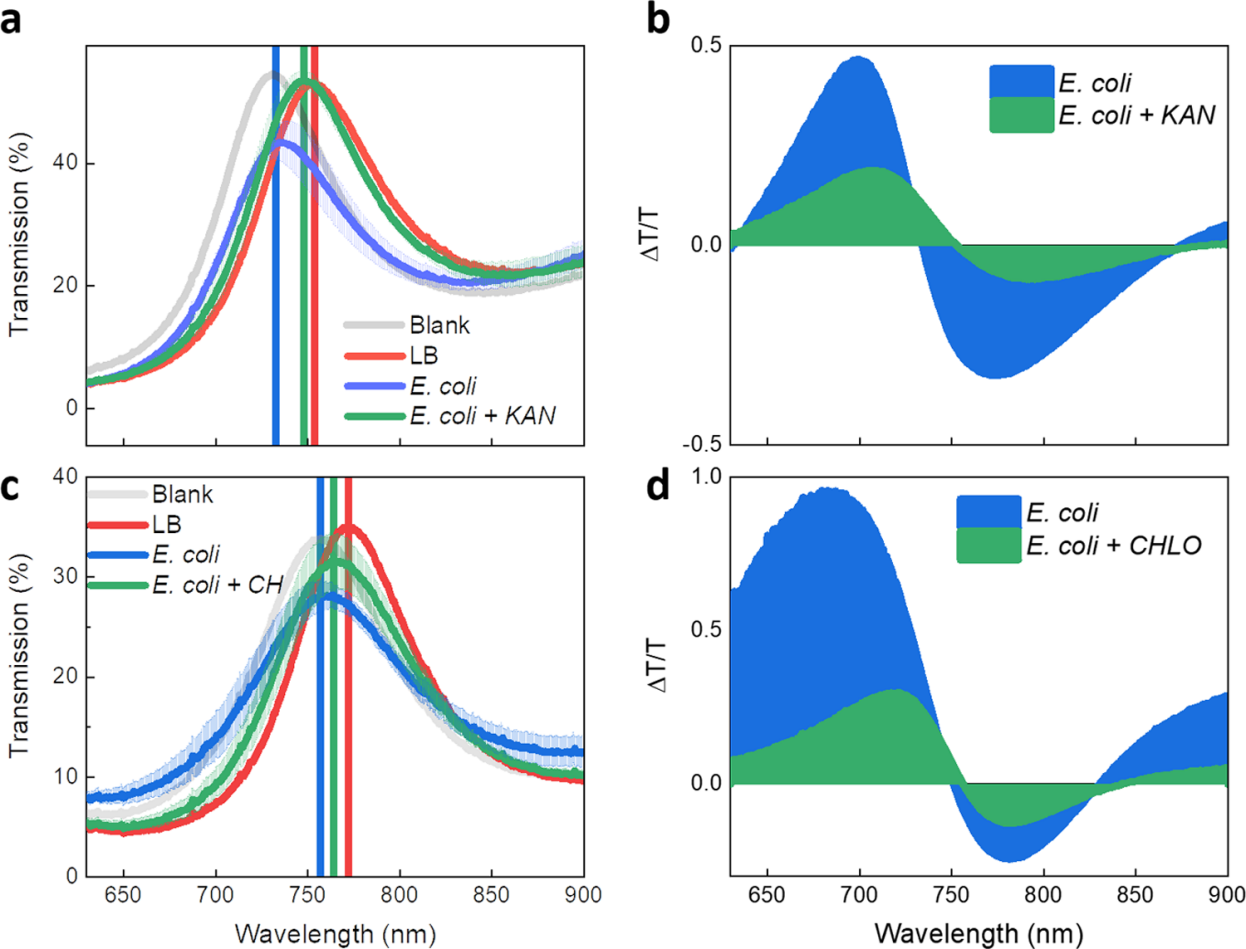
TP resonance is sensible to the proliferative status of bacteria. (a) TP resonance of the device exposed to *Escherichia coli* and *E. coli* treated with kanamycin (KAN, for brevity). (b) Differential transmission spectra (normalized to the transmission of the control measurement, LB), highlighting the modification of the TP resonance upon exposure to *E. coli* and *E. coli* treated with kanamycin. (c) TP resonance of the device exposed to *E. coli* and *E. coli* treated with chloramphenicol (CH for brevity). (d) Differential transmission spectra (normalized to the transmission of the control measurement, LB), highlighting the modification of the TP resonance upon exposure to *E. coli* and *E. coli* treated with chloramphenicol. It is worth reporting a batch-to-batch variability between the devices exposed to KAN-treated and CH-treated bacteria (20% difference in transmission and 25 nm shift in wavelength) that we attribute to the spin-casting technique, consisting of manual sequential deposition of silica and titania layers from colloidal dispersions. This implies that layer thickness and roughness can slightly depend on the deposition conditions (temperature and humidity of the laboratory, as well as on the operator).

This latter biological sample was prepared via the administration of two selected drugs: the antibiotic kanamycin and the bacteriostatic drug chloramphenicol. The former inhibits protein synthesis by compromising ribosome activity, leading eventually to a complete lack of protein turnover,^60^ while the latter permits to achieve the same result by preventing the elongation of the peptide chain on the 50S subunit of ribosomes.^61^ It is thus expected that, in this case, the interaction between bacteria and silver cannot occur effectively due to the excessively low viability of the cell population. These results stem from either the presence of death cells owing to treatment with an antibiotic or from the inhibition of bacterial growth and reproduction brought about by a bacteriostatic drug. Indeed, we observed that nonproliferative bacteria lead to only a 5 nm blue shift and virtually no decrease of the transmission intensity of the TP resonance (Figure 5a). On the other hand, proliferative *E. coli* cells lead to ∼20 nm blue shift and 20% damping of the transmission intensity, in analogy with what was observed in the previous sample batches. These effects are highlighted in the differential spectra (Figure 5b), in which the diagnostic negative signal at 760 nm due to TP blue shift and decrease of transmission results greatly reduced for the device exposed to nonproliferative bacteria.

We then prepared another device batch to evaluate the effect of the chloramphenicol-treated bacteria on the Tamm mode (Figure 5c). Here, we observe that the Tamm resonance overlaps to a lesser extent with the control curve if compared with the case of kanamycin, indicating that such a drug is likely less effective than kanamycin in inhibiting the capability of silver to interact with bacteria. This is also underlined by the diagnostic negative signal lying at around 760 nm (Figure 5d), which is appreciably less damped than in the case of kanamycin. Based on such differential curves, one can conceive a simple ratiometric sensing approach, in which the differential transmission at 760 nm measured with a stable light source (i.e., laser) can be taken as an indicator of the proliferative activity of bacteria and, thus, of their susceptibility against external stressors.

## 4 Conclusions

In this paper, we have demonstrated that TP resonance is sensitive to both the presence of bacteria and, interestingly, to their proliferative status, as highlighted by their interaction with silver. Specifically, we fabricated DBRs via facile spin-casting deposition from colloidal dispersion of silica/titania nanoparticles and capped the dielectric mirror with a nanostructured layer of silver. The corrugation at the nanoscale did not hamper the occurrence of a well-defined TP resonance at the low-energy side of the PBG (photonic bandgap). In fact, it permitted access to the TP field from the outside, as observed in a recent publication^34^ and confirmed by our electro-doping experiments. The modification of the silver optical properties brought about by the model bacterium *E. coli*,^13,14,51^ which can be interpreted within the scheme of bacterial-driven oxidative dissolution of the metal, are translated into the modulation of the TP resonance mode intensity (decrease of transmission) and spectral position (blue shift).

Electrodoping experiments and theoretical modeling confirmed that such effects could be connected to an excess of electron density in the metal layer owing to the removal of positive ions from its lattice. Finally, as a case study, we tested the capability of our TP device to discriminate between proliferative and nonproliferative bacteria. Taken together, these data can pave the way to the introduction of optical sensors whose simple readout can be exploited for monitoring the metabolic status of bacteria, with a view to building drug testing platforms.

## Supporting Information Available

Optimization of the metal layer for the development of the TP resonance; particle size analysis; bacterial growth inhibition test; effect of *Salmonella enterica* exposure on TP devices; sketch of the experimental apparatus used for electrodoping experiments; and Supporting details on the calculation of the optical features for TP devices (PDF)
