# Supplementary material for: Tamm Plasmon Resonance as Optical Fingerprint of Silver/Bacteria Interaction
Source: ACS Appl Mater Interfaces. 2023 Jun 1;15(23):27750–8. doi: 10.1021/acsami.3c05473 (PMC10273179; doi:10.1021/acsami.3c05473)
Supplement: Supplementary file 1 — am3c05473_si_001.pdf [file am3c05473_si_001.pdf]

Supporting Information

Tamm Plasmon Resonance as Optical Fingerprint of Silver/Bacteria Interaction

Simone Normani[1], Pietro Bertolotti[1,2], Francesco Bisio[3], Michele Magnozzi[4], Francesco Federico Carboni[1], Samuele Filattiera[1], Sara Perotto[1], Fabio Marangi[1,2], Guglielmo Lanzani[1,5], Francesco Scotognella[5] and Giuseppe Maria Paternò[1,5]*

[1]Center for Nano Science and Technology@PoliMi, Istituto Italiano di Tecnologia, Via Giovanni Pascoli, 70/3, 20133 Milano, Italy

[2]Biomedical Engineering Department, Politecnico di Milano, Piazza Leonardo Da Vinci, 32, 20133 Milano, Italy

[3]SuPerconducting and other INnovative materials and devices institute (SPIN), Consiglio Nazionale delle Ricerche (CNR), Corso F.M. Perrone 24, 16152 Genova, Italy

[4]Dipartimento di Fisica, Università di Genova, via Dodecaneso 33, 16146 Genova, Italy

[5]Physics Department, Politecnico di Milano, Piazza Leonardo Da Vinci, 32, 20133 Milano, Italy

*Authors to whom correspondence should be addressed: giuseppemaria.paterno@polimi.it

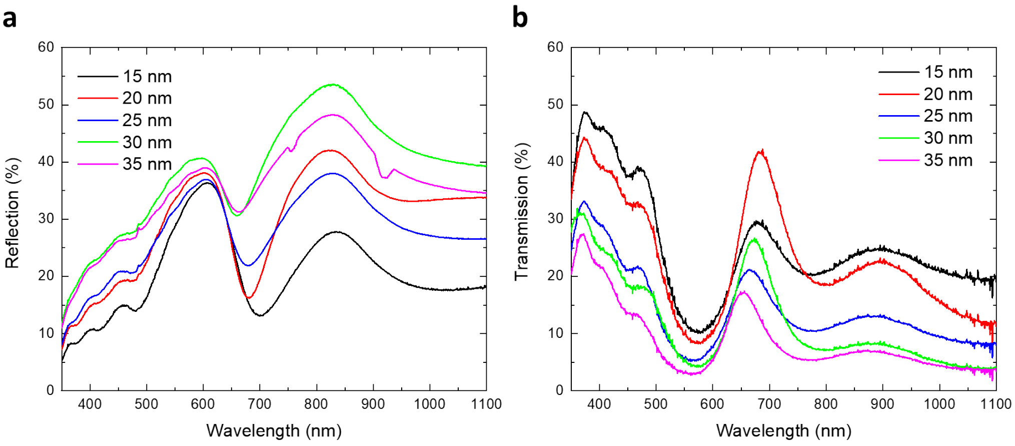

Figure S1. Optimisation of the metal layer for the development of the TP resonance. a. reflection and **b**. transmission of the TP device with different Ag layer thicknesses, keeping constant the DBR parameters.

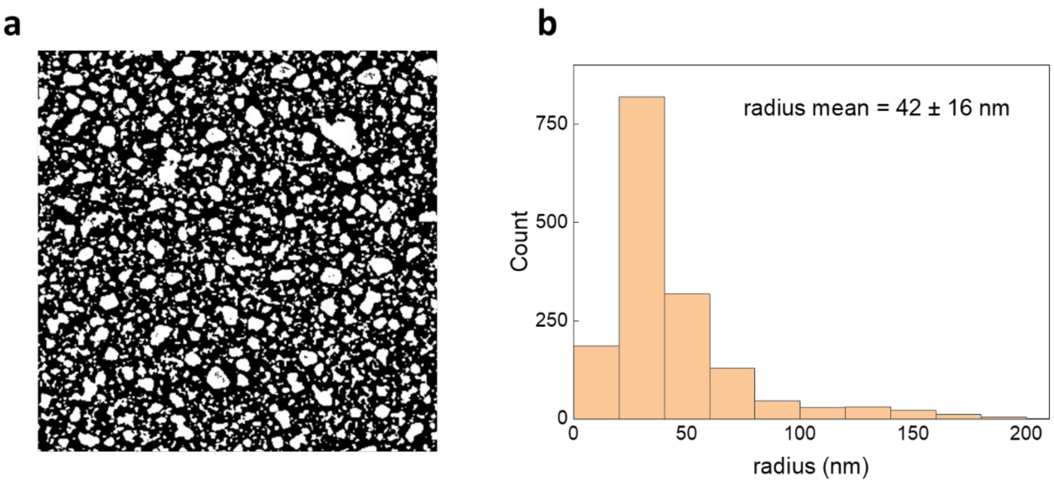

Figure S2. Particle size analysis carried out via SEM. a. representative region from which we extract the particle size distribution. **b.** Particle size distribution. The analysis was carried out by using the software ImageJ.

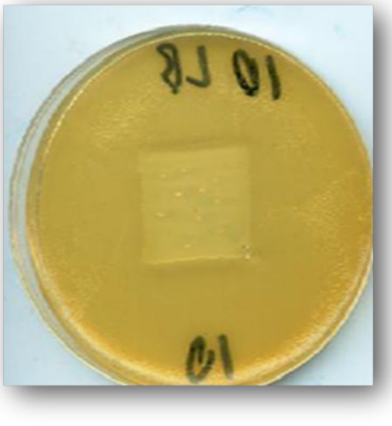

Figure S3. Zone of inhibition left behind from our TP device on a Agar Plate contaminated with *E. coli* cells. This confirms the biocidal activity and, thus, the bio-responsivity of the capping silver layer.

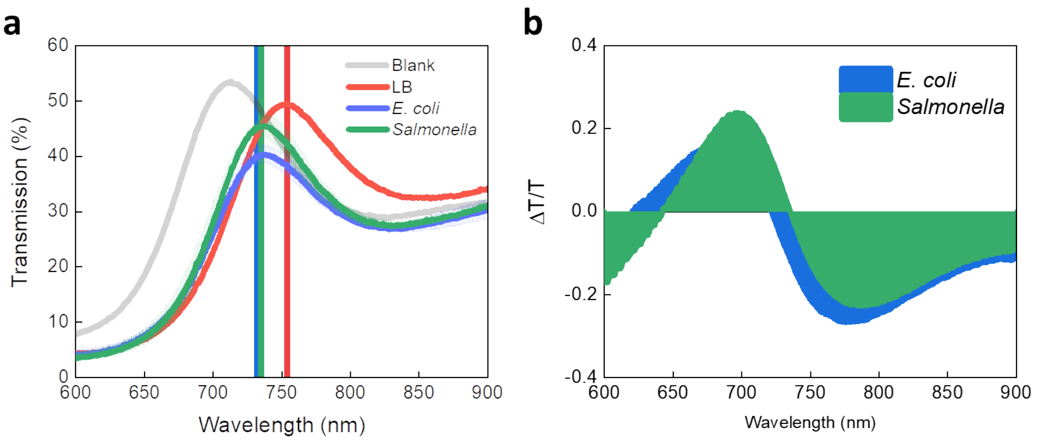

Figure S4. The effect of *Salmonella enterica* exposure on TP-devices. a. Transmission of the TP-resonance upon contamination with E. coli and Salmonella. **b**. differential spectrum (ΔT/T) calculated as $(\boldsymbol{T_{bacteria}} - \boldsymbol{T_{LB}})/\boldsymbol{T_{LB}}$, which highlight the modification of the spectral response (both shifts and changes in transmission) of the TP resonance when exposed to bacteria. The two differential spectra almost overlaps, indicating that the TP resonance cannot discriminate between bacteria exhibiting the same cell wall (Gram-negative).

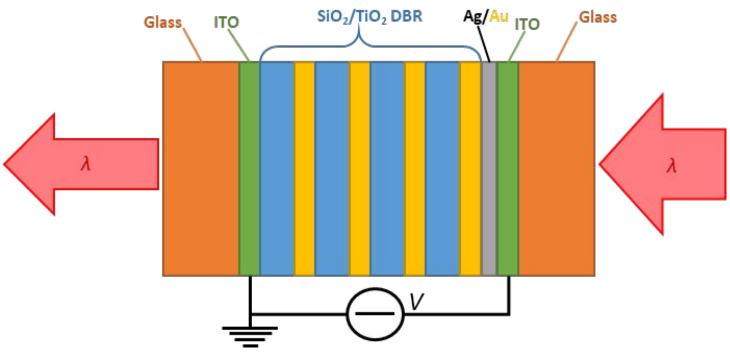

Figure S5. Sketch of the experimental apparatus used for electrodoping experiments. For these experiments, we followed virtually the same procedure that we employed in reference.[1]

Optical simulation of the TP device

In order to rationalize the experimental observations, we performed a set of simulations of the optical response of the DBRs (**Fig. S6**). The simulations were done for pristine DBRs, before and after the deposition of the Ag layer, and then the presence of the LB and the bacteria was effectively simulated. In Figure S6, top panel, we report the experimental reflectance curve (green markers) of a DBR, along with the simulated curve corresponding to the best-fit morphological characteristics of the silica and titania porous layers. The best fit was achieved with a layer thickness of 110 nm (71 nm) and a void fraction of 24% (21%) for the silica (titania) layers. The model, correctly reproduces all the main spectral features of the reflectivity, with only minor intensity discrepancy. The optical response of the DBR with an additional Ag layer was modelled by adding a further dielectric layer on top of the previously-fitted DBR. The dielectric function of the discontinuous Ag layer on top of the DBR was modelled as a linear effective layer, i.e. a linear combination of the dielectric function of Ag (taken

from reference[2]) and the one of the ambient (voids in this case). The layer thickness and the void fraction were left as free parameters. In Figure S6, bottom panel, we report the experimental reflectance (blue markers) along with the best fit (red line), corresponding to a film thickness of 33 nm and a void fraction just below 50 %. The appearance of the reflectance dip corresponding to the Tamm plasmon is correctly reproduced.

Modelling the optical response of the system following the interaction of the system with the bacteria requires some simplifications. We will examine the general trends as a function of individual variations of the system parameters that mimic the interaction with bacteria. The factors that may affect the optical response are: i) the immersion of the system in the LB broth, ii) the re-arrangement of the Ag layer and iii) the dispersion of Ag ions in the bacteria. The simulated curves are reported in Figure S6b. The black curve represents the best fit for the DBR+Ag system in air. The interaction with the LB broth can be reproduced considering the ambient to be a transparent medium with n=1.33 refractive index (including the voids in the Ag layer). Applying this change (red line in Figure S6b), we observe a general slight decrease of reflectance, and no significant spectral shifts of the Tamm plasmon.

The variation of morphology of Ag can be modelled by toggling the Ag fraction in the effective layer, while varying the film thickness to keep the effective amount of Ag constant. This is done under the assumption that the system is immersed in the LB. The effect of this modification on the Tamm plasmon is a red shift when the Ag content decreases (and the thickness correspondingly increases) and vice versa. To give an idea, if the Ag content in the effective layer decreases from 50% to 40%, and the thickness increases to 41 nm (to keep the total Ag constant), then the Tamm plasmon redshifts by 7 nm (compare green line with red line in Fig. S6b).

The dispersion of Ag ions in the bacteria can be represented by an increase in the free-electron density of Ag. From the optical point of view, if this change is moderate, it can be represented by a corresponding variation of the free-electron contribution to the dielectric function (the so-called Drude term). To give a quantitative estimation, augmenting the free-electron contribution by 5% led to a Tamm-plasmon blueshift of around 3 nm (compare blue line with red line in Fig. S6b). The simulations prove that there is a number of factors that affect the spectral characteristics of the Tamm plasmon. The experimental behavior recorded originates from a weighed superposition of these factors. The prevalence of redshifts over blueshifts in different experimental stages can be ascribed to the corresponding prevalence of each of the above-described factors on one another. We thus conclude that in our case, the accumulation of electron charge density would represent the most probable scenario.

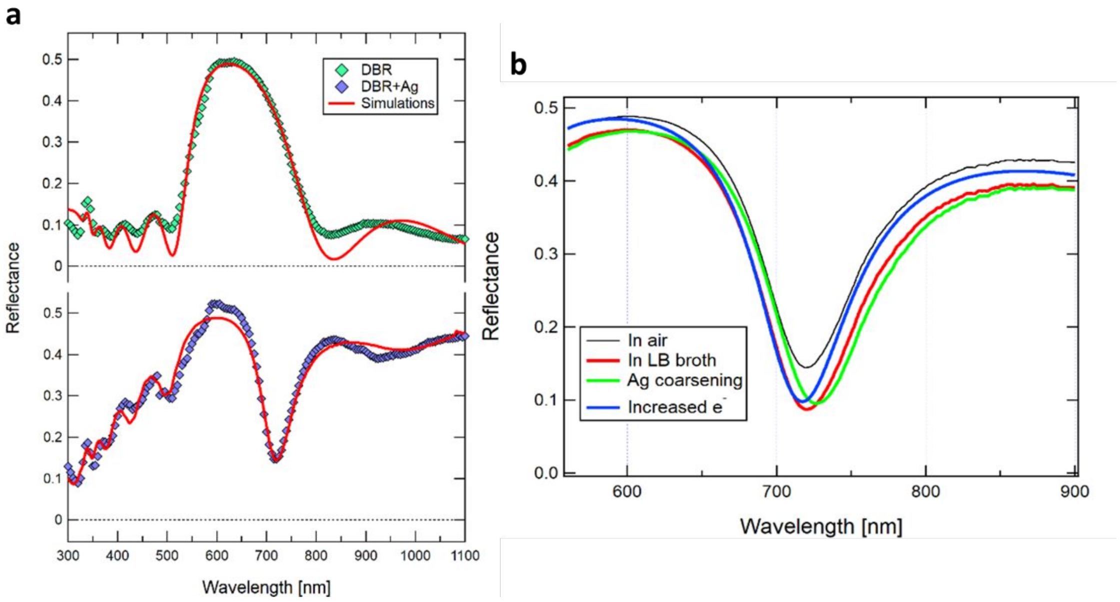

Figure S6. Optical simulation of the TP device. a. top panel: experimental reflectance of the DBR (green markers) and best fit (red line). Bottom panel: experimental reflectance of the DBR+Ag system (blue markers) along with its best fit (red line). **b**. Simulated optical response of the DBR+Ag upon interaction with bacteria. Black line: DBR+Ag in air. Red line: DBR+Ag immersed in the LB broth. Green line: effect of Ag coarsening in LB broth. Blue line: effect of augmenting the free-carrier density in Ag, in LB broth (see text for details).

REFERENCES

(1) Moscardi, L.; Paternò, G. M.; Chiasera, A.; Sorrentino, R.; Marangi, F.; Kriegel, I.; Lanzani, G.; Scotognella, F. Electro-Responsivity in Electrolyte-Free and Solution Processed Bragg Stacks. *J. Mater. Chem. C* **2020**, *8* (37), 13019–13024. https://doi.org/10.1039/d0tc02437f.

(2) Ferrera, M.; Magnozzi, M.; Bisio, F.; Canepa, M. Temperature-Dependent Permittivity of Silver and Implications for Thermoplasmonics. *Phys. Rev. Mater.* **2019**, *3* (10), 105201. https://doi.org/10.1103/PhysRevMaterials.3.105201.
